# Mitochondrial encephalomyopathy with lactic acidosis and stroke-like episodes with an *MT-TL1 m.3243A>G* point mutation: Neuroradiological features and their implications for underlying pathogenesis

**DOI:** 10.3389/fnins.2022.1028762

**Published:** 2023-01-06

**Authors:** Helin Zheng, Xuemei Zhang, Lu Tian, Bo Liu, Xiaoya He, Longlun Wang, Shuang Ding, Yi Guo, Jinhua Cai

**Affiliations:** ^1^Ministry of Education Key Laboratory of Child Development and Disorders, Chongqing Key Laboratory of Pediatrics, Department of Radiology, National Clinical Research Center for Child Health and Disorders, Children’s Hospital of Chongqing Medical University, Chongqing, China; ^2^Ministry of Education Key Laboratory of Child Development and Disorders, Chongqing Key Laboratory of Pediatrics, Department of Medical Affairs, National Clinical Research Center for Child Health and Disorders, Children’s Hospital of Chongqing Medical University, Chongqing, China; ^3^Ministry of Education Key Laboratory of Child Development and Disorders, Chongqing Key Laboratory of Pediatrics, Department of Pediatric Neurology, National Clinical Research Center for Child Health and Disorders, Children’s Hospital of Chongqing Medical University, Chongqing, China

**Keywords:** MELAS, *m.3243A>G*, neuroradiological features, stroke-like episodes, polymorphic lesions

## Abstract

**Objective:**

Mitochondrial encephalomyopathy with lactic acidosis and stroke−like episodes (MELAS) is one of the most common inherited mitochondrial disorders. Due to the high clinical and genetic heterogeneity of MELAS, it is still a major challenge for clinicians to accurately diagnose the disease at an early stage. Herein, we evaluated the neuroimaging findings of MELAS with an *m.3243A>G* mutation in *MT−TL1* and analyzed the possible underlying pathogenesis of stroke-like episodes.

**Materials and methods:**

Fifty-nine imaging studies in 24 patients who had a confirmed genetic diagnosis of *m.3243A>G* (*MT-TL1; tRNA*^Leu^) associated with MELAS were reviewed in our case series. The anatomic location, morphological features, signal/intensity characteristics and temporal evolution of lesions were analyzed on magnetic resonance imaging (MRI), and computed tomography (CT) images. The supplying vessels and metabolite content of the lesions were also evaluated by using MR angiography (MRA)/CT angiography (CTA), and MR spectroscopy (MRS), respectively.

**Results:**

The lesions were most commonly located in the posterior brain, with 37 (37/59, 63%) in the occipital lobe, 32 (32/59, 54%) in the parietal lobe, and 30 (30/59, 51%) in the temporal lobe. The signal characteristics of the lesions varied and evolved over time. Bilateral basal ganglia calcifications were found in 6 of 9 (67%) patients who underwent CT. Cerebral and cerebellar atrophy were found in 38/59 (64%) and 40/59 (68%) patients, respectively. Lesion polymorphism was found in 37/59 (63%) studies. MRS showed elevated lactate doublet peaks in 9/10 (90%) cases. MRA or CTA revealed that the lesion-related arteries were slightly dilated compared with those of the contralateral side in 4 of 6 (67%) cases.

**Conclusion:**

The imaging features of MELAS vary depending on the disease stage. Polymorphic lesions in a single imaging examination should be considered a diagnostic clue for MELAS. Stroke-like episodes may be involved in a complex pathogenetic process, including mitochondrial angiopathy, mitochondrial cytopathy, and neuronal excitotoxicity.

## 1. Introduction

Mitochondrial diseases are a group of progressive inherited metabolic diseases characterized by defects in oxidative phosphorylation caused by mutations in nuclear DNA and mitochondrial DNA (mtDNA) genes encoding mitochondrial structural or functional proteins (Gorman et al., 2016). Mitochondrial encephalomyopathy with lactic acidosis and stroke−like episodes (MELAS) is the most common mitochondrial disorders that includes polygenetic, maternally inherited mutations in mtDNA (Sparaco et al., 2003; El-Hattab et al., 2012). It accounts for approximately 15% of all patients with the *m.3243A>G* mutation (Pickett et al., 2018). Approximately 80% of MELAS cases are caused by the *m.3243A>G* mutation in the tRNA leucine (*UUR*) gene (*MT-TL1*) (Lin et al., 2014).

Due to the high heterogeneity of mitochondrial genes (the mutant types and wild types coexist, and the mutation rate and the threshold of disease are different within individuals, organs, and even the same tissues and cells), the same clinical phenotypes might be caused by different mutations, and the same mutation in mitochondria can cause different clinical phenotypes. Thus, MELAS is highly variable and non-specific in its clinical presentation, characterized by multisystem involvement, particularly with organs highly dependent on aerobic metabolism, including the central nervous, musculoskeletal, endocrine, and gastrointestinal systems. The main clinical manifestations of MELAS include headache, vomiting, impaired consciousness, seizures, muscle weakness, visual field defects, deafness, growth retardation, endocrinopathies, dementia, diabetes mellitus, gastrointestinal dysmotility, etc. (Pavlakis et al., 1984; Koenig et al., 2016; Zigman et al., 2021). Generally, MELAS follows a relentlessly progressive course, although not always fatal (Scaglia et al., 2004). Although progress has been achieved in the etiology, pathogenesis, diagnosis, and treatment of MELAS since it was first described in 1984 (Pavlakis et al., 1984), early accurate diagnosis of MELAS is still enormously challenging even for clinicians who are familiar with the clinical manifestations of the disease. The rate of misdiagnosis remains high, especially in the early stages of the disease. At the same time, although the molecular basis of MELAS has been elucidated in the *MT-TL1* mtDNA gene (*m.3243A>G*), the pathogenesis of stroke-like episodes (SLEs) remains controversial. Recently, with the technological development of magnetic resonance imaging (MRI) and computed tomography (CT), neuroimaging has played an increasingly important role in the accurate diagnosis of MELAS and speculation on the pathogenesis of SLEs.

In this study, we retrospectively analyzed the clinical manifestations and neuroimaging findings of MELAS syndrome with an *m.3243A>G* mutation in *MT−TL1*, emphasizing the distribution, signal characteristics and temporal evolution of lesions, and proposing that the significance of polymorphic lesions in the diagnosis of MELAS in a single examination for the first time. Our aims were to discover imaging features for the accurate diagnosis of MELAS, explore the possible underlying pathogenesis of SLEs, and place the pathogenetic process in the context of clinical practice.

## 2. Materials and methods

### 2.1. Ethics statement

The study protocol was approved by the Human Ethics Committee of Children’s Hospital of Chongqing Medical University and was conducted in accordance with the tenets of the Declaration of Helsinki. The need for informed consent from the patients was waived.

### 2.2. Patient selection

Between May 2015 and Jan 2022, a total of 29 pediatric patients with MELAS underwent MR or CT examinations in the Department of Radiology at Children’s Hospital of Chongqing Medical University. The diagnosis was in line with the diagnostic criteria of MELAS in 2002 (Iizuka et al., 2002): (1) The presence of at least one episode of neurologic manifestations compatible with SLEs; (2) demonstration of an acute brain lesion on either CT or MRI corresponding to the neurologic manifestations; (3) elevation of the lactate level in cerebrospinal fluid (CSF); and (4) the presence of ragged-red fibers and vessels with strong reaction to succinate dehydrogenase on muscle biopsies.

**FIGURE 1:**
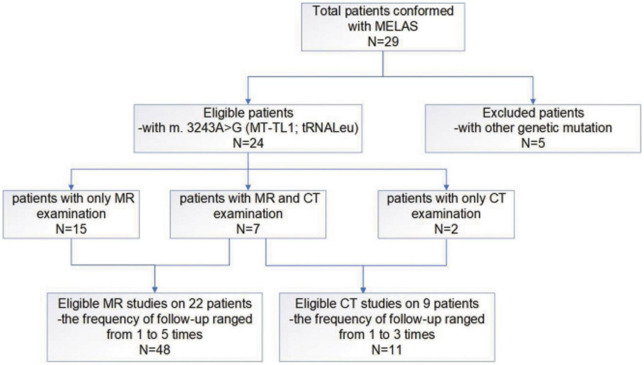
A flow-chart of the entire work procedure.

Since this study mainly focused on MELAS induced by the *m.3243A>G* mutation in *MT-TL1*, five patients with other genetic mutations were excluded from the 29 patients. Hence, data from 24 pediatric patients with MELAS of the *m.3243A>G* mutation in *MT−TL1* (range: 2.3–12 years, median age: 9.25 years, average: 9.05 years ± 2.91 SD; male/female: 10/14) with 48 cerebral MR imaging studies in 22 patients (the frequency of follow-up ranged from 1 to 5 times) and 11 cerebral CT imaging studies (two patients underwent only cerebral CT examination) were included in our case series study (Figure 1).

The clinical data and genetic results were obtained from patients’ medical records, and imaging data were analyzed and recorded based on the imaging records.

### 2.3. Magnetic resonance imaging examinations

Uncooperative subjects were sedated with 0.5 mL/kg 10% chloral hydrate (made at the Children’s Hospital of Chongqing Medical University) administered orally 20 min before MRI examination.

Brain MRI scans were performed on a 3.0-T MRI scanner (Achieva, Philips, Holland) with an eight-channel head coil (SENSE-Head-8). A supine, head-first position was used for all patients. Images were obtained in the axial, sagittal, and coronal planes. The scanning sequences and parameters were as follows: Slice thickness 5 mm, slice gap 1 mm; the field of view (FOV) ranged from 22 to 24 cm covering the whole brain; repetition time = (*TR*) 2,000 ms, echo time (*TE*) = 20 ms, inversion time (*TI*) = 800 ms, for axial T1-weighted imaging (T1WI); *TR* = 3,500 ms, *TE* = 80 ms for axial T2-weighted imaging (T2WI); *TR* = 3,000 ms, *TE* = 80 ms for sagittal T2WI; *TR* = 8,000 ms, *TE* = 125 ms, *TI* = 2,500 ms for axial T2 fluid-attenuated inversion recovery (FLAIR); *TR* = 2,298 ms, *TE* = 87 ms, b factors = 0 and 1,000 or 800 mm^2^/s for diffusion-weighted imaging (DWI). Apparent diffusion coefficient (ADC) maps were automatically calculated from the original DWI data on a workstation (EWS, Philips, Eindhoven, the Netherlands). Three of 22 patients underwent contrast-enhanced MR examination. Gadopentetate dimeglumine (Magnevist, Bayer, Berlin, Germany) was intravenously manually injected at a dose of 0.1 mmol/kg and flushed with the same volume of saline, at the same flow rate when possible. Then, contrast-enhanced and fat-suppressed T1WI of the axial, sagittal and coronal planes was immediately and successively performed with the same parameters as the abovementioned T1WI. Single-voxel ^1^H MR spectroscopy (^1^H-MRS) was performed to separate lactate from lipids by confirming peak inversion using different TEs (144 ms and 35 ms, respectively). Brain MR angiography (MRA) was performed using three-dimensional time of flight (TOF), and the intracerebral artery images were obtained using maximum intensity projection (MIP) reconstruction. The parameters were as follows: *TR* = 25 ms, *TE* = 3.5 ms, 200 slices with a slice thickness of 1.6 mm and FOV of 20 cm.

### 2.4. Computed tomography examinations

Uncooperative subjects were sedated with 0.5 mL/kg 10% chloral hydrate (made at the Children’s Hospital of Chongqing Medical University) administered orally 20 min before CT examination.

Computed tomography images were obtained on a 256-slice CT scanner (Brilliance iCT, Philips, Netherlands). The scanning parameters were as follows: Tube voltage, 100 kilovolts (kV); automatic tube current, from 100 mA to 150 mA; screw pitch, 0.39; and the idose iterative reconstruction algorithm was used for data processing. The scanning range extended from the auditory canthus line up to the top of the skull.

Intravenous contrast non-ionic iohexol (Omnipaque, 350 mg/mL, Amersham Healthcare, Shanghai, China) was injected *via* an antecubital fossa cannula using a double barrel high-pressure syringe (Empower CTA, Bracco Injeneering S. A company, USA) using 1.5–2 ml/kg contrast at 1.5 ml/s followed by 10–15 ml of saline at the same rate. A fluoroscopy trigger was used for the scanning. The trigger zone was set at the center of the common carotid artery at the horizontal level of the third cervical vertebra, with a circular region of interest (ROI). The size of the ROI was 10 mm^2^. The scanning range was from the base of the skull to the superior extent of the calvaria, the scanning direction was from inferior to superior, and the slice thickness was 5 mm.

### 2.5. Image analysis

Fifty-nine imaging studies in 24 MELAS patients with *m.3243A>G* in *MT-TL1* were reviewed. Lesion location, cerebral and/or cerebellar atrophy, signal characteristics, morphological features, and temporal evolution of lesions were analyzed on MR and CT images. Calcification, especially in the basal ganglia, was assessed mainly on CT images. In addition, metabolism of the lesions was evaluated *via* MRS in 12 patients, and the supplying vessels of lesions were also analyzed by using MRA/CT angiography (CTA) in six patients.

In this study, we propose the concept of lesion polymorphism. In a single imaging examination, whether CT or MR, the lesion can be considered polymorphic if it meets more than three of the following conditions: (1) Swelling in the cerebral cortex and subcortical with an increased DWI signal and an increased ADC; (2) swelling in the cerebral cortex and subcortical with an increased DWI signal and a decreased ADC; (3) cortical laminar necrosis with high linear T1 signal intensity; (4) black toenail sign (Whitehead et al., 2017): Focal lesions with an increased signal on T2WI and at least some degree of signal suppression on T2-FLAIR sequences; (5) cerebellar atrophy, cerebral atrophy and/or localized encephalomalacia with gliosis; and (6) calcification of bilateral basal ganglia or/and dentate nuclei of cerebellum.

All studies were reviewed in consensus by two pediatric neuroradiologists with 10–20 years of experience. Although the diagnosis was known, the readers were blinded to the imaging report and clinical data during the review. In the event of disagreement, the two neuroradiologists repeated the double-blind reading along with a third senior neuroradiologist until a consensus was reached.

### 2.6. Statistical analysis

Statistical analyses were performed using IBM SPSS software (version 23, IBM Corporation, Armonk, NY, USA). Demographic, clinical, and CT/MRI data were summarized using the mean ± standard deviation for continuous variables and counts or proportions for categorical variables.

## 3. Results

### 3.1. Clinical features

Table 1 summarizes the demographics, clinical presentations and laboratory results of each patient. All 24 patients had the same genetic pathogenic variant with *MT-TL1 m.3243A>G*. The mothers of six patients (6/24, 25%) had mutations at the same site, and the rest were uncertain. Nine cases (9/24, 37.5%) had a family history, 12 cases (12/24, 50%) had no family history, and the remaining three cases were uncertain. Two patients (2/24, 8.3%) died, one 9 years after onset and the other 3 years after onset. All patients had had at least one SLE; headache occurred in 17 cases (17/24, 70.8%); hirsutism was diagnosed in 16 cases (16/24, 66.7%); episodic visual impairment and hearing impairment occurred in 13 cases (13/24, 54.1%) and 7 cases (7/24, 29.1%), respectively; and cognitive impairment was found in 7 cases (7/24, 29.1%). Blood lactic acid testing was performed in all 24 cases, of which 20 cases (20/24, 83.3%) showed elevated levels of blood lactic acid, and the remaining 4 cases showed normal levels.

**TABLE 1:** Genetic factors, laboratory results, and clinical presentation.

Patient no.	Sex	NMR	NCT	Age^1^ (year)	Clinical presentation:	BLT	TSMSIMOP	Survival results	Family history
1	M	2	0	12	S, Ep, Mi, EVI, H	El	Y	Su	Y
2	F	1	1	6	S, Mi, EVI, H, W	El	N	Su	N
3	F	2	0	6	S, H, Ep	El	N	Su	N
4	F	1	0	9	S, Ep, Mi, H, C	EI	N	Su	Y
5	M	1	1	7	S, Mi, EVI, Ep, H, C	El	N	Su	N
6	F	5	1	10	S, Ep, C	N	N	De	N
7	F	1	0	10	S, Ep, H, HI, Mi, EVI, C	El	N	De	Y
8	F	4	0	11	S, Mi, Ep, H, C, W, EVI, HI	El	N	Su	N
9	M	5	0	5	S, Mi, Ep, EVI, H, HI	EI	N	Su	NS
‘10	F	0	1	8	S, Ep, H, C, Mi	El	Y	Su	Y
11	F	1	1	4	S, Mi, EVI, C, HI, H	El	Y	Su	Y
12	M	4	1	12	S, Mi, C, W, EVI, H	El	Y	Su	Y
13	F	1	0	2	S, W	N	Y	Su	N
14	F	5	3	5	S, Mi, C, W, H	El	N	Su	NS
15	M	0	1	9	S, Ep, C, EVI	N	N	Su	Y
16	M	2	0	6	S, Mi, Ep, EVI, C, H	El	Y	Su	Y
17	F	1	0	8	S, Ep, C	El	N	Su	N
18	F	1	0	9	S, Ep	El	N	Su	N
19	M	3	0	10	S, Ep, Mi, W, EVI, H	El	N	Su	N
20	F	2	1	11	S, C, Ep, H, W, Mi	El	N	Su	N
21	M	1	0	5	S, C, Ep, Mi, H	El	N	Su	Y
22	F	1	0	12	C, Ep, S	El	N	Su	N
23	M	3	0	11	W, S, Mi, HI, EVI	N	N	Su	NS
24	M	1	0	8	C, Ep, Mi, S, HI, EVI	El	N	Su	N

M, male; F, female; N_MR_, numbers of MR examination; N_CT_, numbers of CT examination; Age^1^, age at the time of first neuroimaging examination; HI, hearing impairment; S, stroke-like episode; Ep, epilepsy; C, cognitive impairment; Mi, migraine-like headache; EVI, episodic visual impairment; H, hairy; El, elevated; W, weakness; Y, yes; N, normal; NS, not sure; BLT, blood lactate testing; TSMSIMOP, the same mutation site in the mother of the patient; De, death; Su, survival.

### 3.2. Imaging features

#### 3.2.1. Lesion distribution

As summarized in Table 2, the lesions predominantly involved the posterior brain cortical region. The cortical distribution of lesions was as follows: The occipital lobe was the most commonly affected (37/59, 63%), followed by the parietal lobe (32/59, 54%) and temporal lobe (30/59, 51%), and the frontal lobe (2/59, 3%) was the least commonly affected. The basal ganglia (21/59, 36%) and thalamus (10/59, 26%) were also involved.

**TABLE 2:** Neuroradiological presentation and temporal evolution.

Patient no.	Age^1^ (year)	Time of follow up	MR	The location of the abnormal signal/density	Cerebral atrophy	Cerebellar atrophy	MRS	MRA/CTA	Polymorphism
1–1	12.5		MR	BAG	+	+	ND	ND	+
1–2		3 month	MR	BAG	+	+	ND	ND	+
2–1	6.8		CT	BAG, BF, BP, BO, RT	−	−		ND	−
2–2		5 days	MR	BAG, BO, RT	+	−	+	ND	+
3–1	12		MR	BP, LO	+	−	+	ND	+
3–2		1 month	MR	LP, LO, ***RT, RP***	+	−	ND	ND	+
4	9.2		MR	LT, LP, LO, BAG	−	+	ND	ND	−
5–1	12.8		CT	LT, LP, BO, BAG	−	−		ND	−
5–2		3 days	MR	LT, LP, BO, BAG	+	+	ND	+	+
6–1	11.3		MR	–	+	+	ND	ND	−
6–2		8 month	CT	***RO, RP***	−	−		ND	−
6–3		8 month	MR	***RO, RP, RT***	−	−	ND	ND	−
6–4		9 month	MR	–	+	+	ND	ND	−
6–5		10 month	MR	***LO, LP***	+	+	ND	ND	+
6–6		24 month	MR	***RO, RP, RT***	+	+	ND	+	+
7	10		MR	BAG	+	+	ND	ND	+
8–1	11.5		MR	BAG, BP, LT, BO, LTh, RC	+	+	+	−	+
8–2		1 month	MR	BAG, BP, LT, BO, ***RF***, LTh, RC	+	+	ND	ND	+
8–3		7 month	MR	BAG, BP, LT, BO, ***BF, RT***, LTh, RC	+	+	ND	ND	+
8–4		36 month	MR	***BT, BO, BP***	+	+	ND	ND	+
9–1	5.3		MR	RT, BO, RAG, RTh	−	−	ND	+	−
9–2		1 month	MR	***RT, BO, RAG, RTh***	−	+	ND	ND	+
9–3		3 month	MR	***BO, RP***	−	+	+	ND	−
9–4		24 month	MR	***RF, BAG***	−	+	ND	ND	+
9–5		29 month	MR	***RF, BAG***	+	+	ND	ND	+
10	8.1		CT	BO, BAG	−	−		ND	+
11–1	4.6		MR	–	−	−	−	ND	−
11–2		6 days	CT	BAG	−	−		−	−
12–1	12.1		MR	LT, LP, LO	−	+	ND	ND	+
12–2		7 days	MR	LT, LP, LO, ***LTh***	−	+	+	ND	+
12–3		9 days	CT	LT, LP, LO	−	−		ND	−
12–4		3 month	MR	***LT, LP, LO, LTh***	+	+	ND	ND	+
13	2.3		MR	–	−	−	ND	ND	−
14–1	5.8		MR	–	−	−	ND	ND	−
14–2		9 month	MR	–	−	−	ND	ND	−
14–3		12 month	CT	–	+	+	ND	ND	−
14–4		36 month	MR	BP, BO	+	+	ND	ND	+
14–5		54 month	MR	***BAG***	+	+	+	ND	+
14–6		58 month	MR	***BT, BP, LO, LTh***, BAG	+	+	ND	ND	+
14–7		60 month	CT	BAG	+	+	ND	ND	+
14–8		61 month	CT	BAG	+	+	ND	ND	+
15	9.3		CT	BAG, BDN, BO	+	−		ND	+
16–1	6.8		MR	BT, LO	+	+	ND	ND	+
16–2		5 month	MR	BT, BF, BP, BAG	+	+	ND	ND	+
17	8.8		MR	RT, RO, RP, RTh	+	+	ND	ND	+
18	9		MR	–	−	−	ND	ND	−
19–1	10.2		MR	–	−	−	ND	ND	−
19–2		9 month	MR	–	−	−	ND	ND	−
19–3		13 month	MR	RO, RP	+	+	+	ND	+
19–4		44 month	MR	***RO, LO***	+	+	ND	ND	+
20–1	11.4		CT	–	+	+	ND	ND	−
20–2		1 days	MR	–	+	+	ND	ND	−
20–3		20 month	MR	LO, LP, LTh	+	+	ND	ND	−
21	5.4		MR	BF, BT, BO, BP, BAG	+	+	ND	ND	+
22	12.5		MR	DO, DP, LT	+	+	+	ND	+
23–1	11.2		MR	RT, RP, RO	+	+	+	ND	+
23–2		4 month	MR	***RT, RP, RO, BAG***	+	+	ND	+	+
23–3		12 month	MR	***RT, RP, RO, BAG***	+	+	ND	ND	+
24	8.4		MR	BO, BP	+	+	ND	ND	+

Age^1^, age at the time of first neuroimaging examination AG, basal ganglia; F, frontal lobe; T, temporal lobe; P, parietal lobe; O, occipital lobe; B, bilateral; R, right; L, left; Th, thalamus; C, cerebellum; DN, dentate nucleus.

Italic and bold text indicates that the lesion signal or site changed compared to the previous timepoint.

#### 3.2.2. Lesion signal features in temporal evolution

**FIGURE 2:**
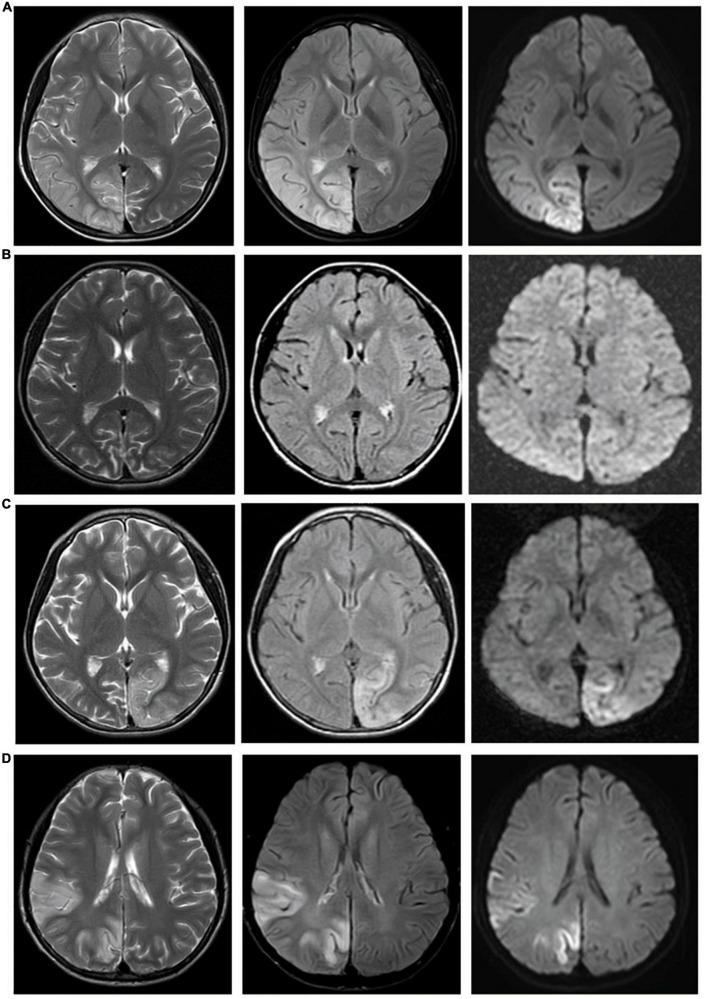
Lesions migrate over time. Case 6: Four MR examinations were conducted successively in a 10-year-old girl with MELAS from 2014 to 2016. **(A)** Abnormal signal in the right temporal, parietal, and occipital lobes. **(B)** Forty-eight days later, MR showed cerebral atrophy, and the abnormal signal in the right temporal, parietal, and occipital lobes disappeared. **(C)** Eighty-seven days later, a new lesion appeared in the left parietal and occipital lobes. **(D)** More than 2 years later, lesions in the left parietal and occipital lobes appeared, and new lesions appeared in the right temporal, parietal, and occipital lobes.

**FIGURE 3:**
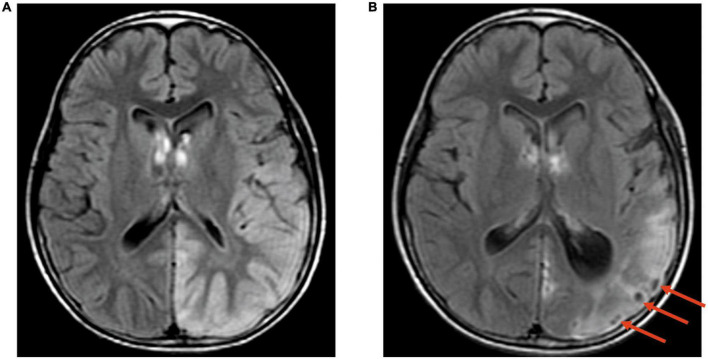
The black toenail sign. Case 12, A 12-year-old boy with MELAS. **(A)** Transverse FLAIR images. Hyperintensity in the left temporoparietal occipital lobe; **(B)** Transverse FLAIR images. The same patient was examined 3 months later again. Cerebral atrophy was observed in the left temporoparietal occipital lobe with the “black toenail” sign (shown by the red arrow) on the top of the gyrus.

The signal characteristics of the lesions varied, mainly depending on the different stages of the disease. During the acute stage, the main finding was swelling of the cortex, which showed signal features similar to those of acute cerebral infarction but not restricted to arterial territories and migrated over time. Six of the 18 patients (33%) with lesions demonstrating acute diffusion restriction on MR images were followed up 1–5 times, and the lesion location, extent and signal changed to various degrees (Figure 2). During the subacute phase, gyriform intracortical T1 hyperintensity with T2 hypointensity was seen on MRI. The black toenail sign (Figure 3) was observed in 9 of 26 (35%) subacute phase studies. During the chronic period, brain volume loss and encephalomalacia with gliosis were typical MR features.

#### 3.2.3. Cerebral and/or cerebellar atrophy

For all 59 imaging studies, cerebral atrophy occurred in 38 (64%), and cerebellar atrophy occurred in 40 (68%). Some patients with MELAS showed no abnormal findings on routine MR except cerebral and/or cerebellar atrophy. Five of 12 (42%) cases without abnormal signals showed brain atrophy.

#### 3.2.4. Lesion polymorphism

**FIGURE 4:**
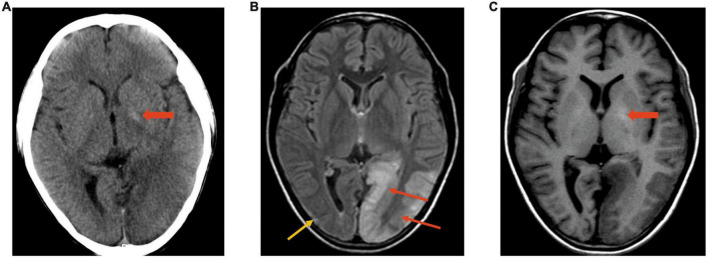
Polymorphism of lesions. Case 5, A 12-year-old boy with MELAS. **(A)** CT image. Density decreases in the left occipital lobe; calcification is slightly evident on the left (wide arrow). **(B,C)** MRI was performed after 3 days in the same boy. FLAIR **(B)** showed polymorphism of the lesion: Cortex swelling on the left occipital (red arrows) and cortex atrophy with gliosis (yellow arrow) on the right occipital. T1WI **(C)** showed hyperintensity in the bilateral basal ganglia and hypointensity in the left occipital region.

In this study, we propose the concept of lesion polymorphism, which refers to the spatial diversity of lesions in a single neuroimaging examination. Forty of 59 (68%) studies showed polymorphisms of lesions in our cohort, which might show cortex swelling, cortex atrophy with gliosis in different regions and calcification of the bilateral basal ganglia (Figure 4).

#### 3.2.5. Other imaging features

**FIGURE 5:**
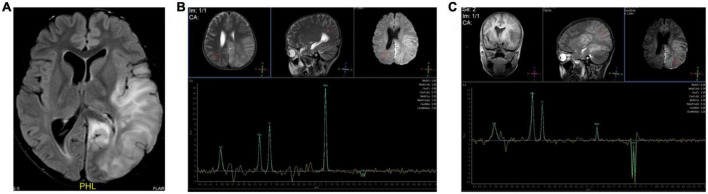
Elevated lactate doublet peaks on MRS. Case 12, A 12-year-old boy with MELAS. **(A)** Transverse FLAIR images showed cortical swelling in the left temporoparietal occipital lobe. **(B,C)** MRS showed elevated lactate doublet peaks in both acutely affected and unaffected regions; however, it was more obvious on the left.

MR spectroscopy assessment was performed in 10 patients, in which 9 (90%) cases demonstrated elevated lactate doublet peaks in both acutely affected and unaffected regions of subcortical white matter accompanied by a decrease in N-acetyl-L-aspartic acid (NAA) (Figure 5).

**FIGURE 6:**
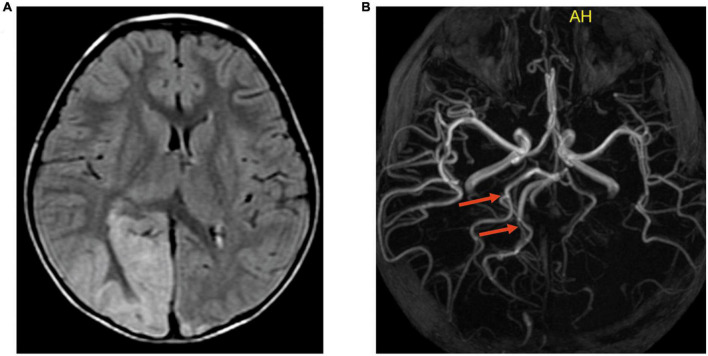
Slightly dilated cerebral artery and the branches relevant to the lesion on MRA. Case 9, A 5-year-old boy with MELAS. **(A)** Transverse FLAIR images showed cortical swelling in the right occipital lobe. **(B)** MRA showed that the right posterior cerebral artery and the branches relevant to the lesion (indicated by the red arrows) were slightly dilated compared with the left.

Brain MRA and CTA were performed in 5 and 1 patients, respectively. The relevant arteries of the lesion were slightly dilated compared with those of the contralateral side in 4/6 (67%) cases (Figure 6).

## 4. Discussion

In this study, we reviewed a group of MELAS case series in children and identified several imaging characteristics, including predominant involvement of the posterior brain cortical region, varying signal characteristics over time, calcification of the bilateral basal ganglia, cerebral and/or cerebellar atrophy, elevated lactate doublet peaks in both acutely affected and unaffected regions, and slight dilation of the relevant arteries. These findings have implications for the early and accurate diagnosis of MELAS. In addition, we propose the novel concept of lesion polymorphism, which features a diversity of lesion morphology and signals and reflects the progression of the disease. If polymorphic lesions are detected in a single imaging examination, the possibility of MELAS should be considered.

Since MELAS is a progressive disease, it often shows various MRI features in different disease stages. In the acute stage, cerebral edema is commonly observed, often involving the occipital or posterior parietal cortex. Cerebral edema can be cytotoxic or vasogenic. In this group of cases, some cortical edema developed into laminar necrosis and brain atrophy, while the other cortical edema disappeared without remaining lesions in a short time. We speculated that it was related to the type of edema (cytotoxic or vasogenic). However, due to the retrospective nature of the analysis, we did not regularly follow up all the children. This required us to better design research routes in the future to find biological markers for predicting lesion evolution in the short term.

With disease progression, the lesions are characterized by laminar necrosis, which often indicates that the lesion is irreversible. Laminar necrosis often showed linear T1 hyperintensity along the gyri. The black toenail sign is specific for indicating gyrus necrosis, which was detected in 35% of subacute cases. When the disease enters the chronic stage, brain atrophy and calcification of the bilateral basal ganglia are common features of MELAS. Lesions at different stages can be presented on a single image, which we call polymorphic lesions. This sign occurred in 40/59 (68%) cases and was relatively specific to MELAS. The previous MELAS-related literature (Khandwala et al., 2018; Tetsuka et al., 2021) emphasized the importance of lesion migration, which needs to be confirmed by follow-up and possibly causes delays in the diagnosis and treatment of disease. Polymorphic lesions, however, can be observed on one single radiologic examination and serve as a diagnostic clue for MELAS.

MR spectroscopy is a useful technique for diagnosing MELAS because it can assess brain metabolites (Feng et al., 2006; Li X. et al., 2018). The lactate levels increased not only in cerebral tissues (the sites with and without the lesion) but also in blood and CSF (Feng et al., 2006), which confirms a level of metabolic derangement (Goodfellow et al., 2012; Chen H. et al., 2020). Ten cases in our study were performed by MRS, and 9 cases showed elevated lactate double peaks in acutely affected and/or unaffected regions. Unfortunately, lactic acid peaks were not measured in CSF in any of our cases. In future prospective studies, we should measure lactate peaks in CSF to better evaluate MELAS.

Computed tomography has limited value in the diagnosis of MELAS, apart from revealing calcifications in the bilateral basal ganglia. According to Majamaa et al. (1998) report, basal ganglia calcification occurred in up to 13% of MELAS and was possibly attributed to dystrophic calcification. In our series, 6 cases showed bilateral basal ganglia calcifications in 9 cases who underwent CT. Basal ganglia calcification is a common incidental finding in healthy adults but is rarely encountered in normal children and is often suggestive of a genetic metabolic disorder. This sign, while not specific to MELAS, can serve as a cue for clinicians to consider the possibility of Melas in combination with other imaging features and clinical manifestations, especially in younger children.

To date, although the precise pathophysiology of SLEs with MELAS has not been completely elucidated, three possible hypotheses for these episodes are as follows: (1) mitochondrial angiopathy (vascular hypothesis) (Iizuka and Sakai, 2005); (2) mitochondrial cytopathy theories caused by an oxidative phosphorylation defect (Iizuka and Sakai, 2005); and (3) non-ischemic neurovascular events initiated by neuronal hyperexcitability (Iizuka et al., 2002), including neuron–astrocyte uncoupling (Mir et al., 2014).

The theory of mitochondrial angiopathy is the concept of vascular caliber alterations and extravasation caused by mitochondrial abnormalities inside vascular smooth muscle cells and vascular endothelial cells on the cerebral surface arterioles and soft membrane arterioles, which result in angioedema. The angioedema on MRI showed an increased DWI signal and an increased ADC value. Twelve cases in our cohort did so. Recently, some studies detected local vasodilation on MRA and hyperperfusion on ASL in some lesions of SLEs (Li X. et al., 2018, Li Y. et al., 2018), which was not consistent with vascular obstruction. In our study, the supplying vessel of the lesion was slightly dilated compared with those of the contralateral side in 4 of 6 cases that received MRA or CTA, which was consistent with the above reports. However, cases of cytotoxic edema in MELAS are also not uncommon. Thus, the mitochondrial angiopathy pathogenesis of SLEs remains controversial. Mitochondrial cytopathy is the theory of intracellular metabolic disorders caused by mitochondrial dysfunction (Iizuka and Sakai, 2005), showing the highly specific distribution of acute lesions to cortical areas of higher neuronal density and metabolic demand. The primary visual cortex located in the occipital lobe has one of the highest metabolic demands (Wong-Riley, 2010) and highest neuronal density (Turner et al., 2016) of all cortical regions. The priority distribution of lesions in the posterior cortical regions has been well described in previous reports of MELAS (Bi et al., 2006; Cai et al., 2016; Hongo et al., 2019), which was consistent with the results of our study. Furthermore, laminar cortical necrosis with sparing of deep white matter (Valanne et al., 1996; Whitehead et al., 2017; Bhatia et al., 2020) and the black toenail sign with an iso-intensity line on its surface demonstrated that the layers of the cerebral cortex with high oxygen demand (especially layers 4–6) are readily affected (Finsterer, 2009) by MELAS. Given these considerations, mitochondrial cytopathic theory may be favored. In addition, SLEs can result in the enhancement of neuron excitability, leading to lesions expanding to the surrounding cerebral cortex. Three patients in our short-term follow-up group (less than 1 month) developed the same manifestation. In MELAS, ATP deficiency can lead to dysfunction of astrocytes, insufficient absorption of glutamine and potassium by astrocytes, and excessive levels of glutamine and potassium in the synaptic cleft lead to cerebrovascular dilatation and increased cerebral blood flow, but neurons cannot produce sufficient ATP, and the metabolism cannot keep up, resulting in an intracellular metabolic imbalance and damage to the cerebral cortex due to excitotoxicity (Attwell et al., 2010; Chen J. et al., 2020). In view of the spread of lesions beyond the arterial region, SLEs are more likely to be caused by neuronal hyperexcitability rather than from mitochondrial angiopathy. Recently, more and more researches proposed that neuronal hyperexcitability can better explain the stroke-like lesions than mitochondrial angiopathy (Ng et al., 2019; Orsucci et al., 2022). In short, changes in cerebral blood flow, oxygen metabolism and neuronal excitability occur during SLEs, resulting in abnormal changes in characteristic distribution patterns and evolution regularity. We speculated that the pathogenesis of SLEs in MELAS is the result of a combination of multiple factors and multiple pathways, although neuronal hyperexcitability may play a major role. MELAS and acute ischemic stroke (AIS) appear to be similar on MRI. Both showed high intensity on DWI and elevated lactate levels. In general, the age of onset of MELAS is younger than that of AIS. In addition, the lesions in MELAS are not confined to the territory of a single major artery; in contrast, AIS lesions coincide with the major artery supplying area. Moreover, studies have shown hyperperfusion in MELAS patients compared with perfusion defects or hypoperfusion in AIS (Rodan et al., 2015; Li X. et al., 2018, Li Y. et al., 2018; Zhang et al., 2020). Of course, MELAS also needs to be differentiated from viral encephalitis and brain tumors, especially in young patients. In this study, polymorphic lesions in a single imaging examination was a representative neuroradiological sign of MELAS, which rarely occurs in other diseases. So we believe that this sign can at least be used as a clue for early diagnosis of MELAS, and may be helpful in differentiating MELAS from other diseases.

Our study is limited by its retrospective nature. The imaging modality, parameters, interval time and frequency of follow-up were not uniform, which could lead to a bias in imaging analysis. MRS and MRA were performed only in some cases. Meanwhile, some of the images in our study may not be of high quality, which may affect the diagnosis of the disease. In this case, it is possible to improve image quality by applying the image processor mentioned in the literatures (Khalid et al., 2014; Verbiest et al., 2014; Zhou et al., 2014). In future studies, multiple imaging modalities, including MRS, MRA, and DWI, as well as advanced image processing methods should be performed to deeply investigate the imaging features and pathological mechanism of the disease.

## 5. Conclusion

The imaging manifestations of MELAS depend on the stage of the disease. Cerebral vasogenic or cytotoxic edema is commonly observed in the acute stage. With disease progression, laminar necrosis might be observed. Brain atrophy and calcification of the bilateral basal ganglia are features of MELAS in the chronic stage. In this article, we proposed that polymorphic lesions on a single radiologic examination can serve as an important clue for the early and accurate diagnosis of MELAS. The mechanism of SLEs in MELAS may involve a wide range of factors, comprising mitochondrial angiopathy, mitochondrial cytopathy, neuronal hyperexcitability, and others.

## Data availability statement

The original contributions presented in this study are included in the article/supplementary material, further inquiries can be directed to the corresponding authors.

## Ethics statement

The studies involving human participants were reviewed and approved by the Human Ethics Committee of Children’s Hospital of Chongqing Medical University. Written informed consent from the participants or their legal guardian/next of kin was not required to participate in this study in accordance with the national legislation and the institutional requirements.

## Author contributions

HZ, XZ, YG, and JC designed the study. HZ and JC wrote the manuscript. XZ, LT, and SD analyzed the data. BL, XH, and LW performed the experiments. All authors contributed to the article and approved the submitted version.
